# Accuracy of Declared Salt Content on Traffic Light Labelling of Nuts and Seeds in Isfahan, Iran

**DOI:** 10.34172/jrhs.2024.151

**Published:** 2024-06-01

**Authors:** Fatemeh Shirani, Seyedeh Mahsa Khodaei, Mojtba Akbari, Razieh Eshaghi, Mansour Siavash, Zahra Esfandiari

**Affiliations:** ^1^Isfahan Endocrine and Metabolism Research Center, Isfahan University of Medical Sciences, Isfahan, Iran; ^2^Nutrition and Food Security Research Center, Department of Food Science and Technology, School of Nutrition and Food Science, Isfahan University of Medical Sciences, Isfahan, Iran

**Keywords:** Sodium, Salt, Nuts, Seeds, Traffic light, Food labelling, Iran

## Abstract

**Background:** Regarding the importance of the prevention of non-communicable diseases (NCDs) and higher consumption of salt among the Iranian population than the level recommended by the World Health Organization, the aim of this study was to evaluate the accuracy of the salt mentioned in the traffic light labelling of nuts and seeds.

**Study Design:** A cross-sectional study.

**Methods:** A total of 53 packaged nuts and seeds, including 7, 8, 9, 9, 10, and 10 samples of pumpkin, pistachios, almond, sunflower, peanut, and watermelon nuts and seeds, respectively, with traffic light labelling, were randomly purchased from several local markets in Isfahan, Iran. The amount of sodium was measured by the inductively coupled plasma-optical emission spectroscopy technique and then multiplied by 2.5 to achieve the amount of salt.

**Results:** Varying levels of traffic light labeling value accuracy were observed in most of the samples. In the almond, pistachio, peanut, and watermelon groups, the average amount of laboratory value had a statistically significant difference with the label value (*P*<0.05).

**Conclusion:** The results demonstrated that the salt content of 82% of the studied samples had discrepancies with the values stated on the traffic light labelling. The presentation of an accurate amount of salt content is essential for promoting healthy eating habits and enabling individuals to make informed choices about their diet. It is recommended that regulatory authorities should review labelling guidelines and enforce stricter compliance to ensure accurate representation of salt content on packaged foods.

## Background

 Non-communicable diseases (NCDs), including heart disease, stroke, diabetes, cancer, and chronic respiratory disease, are the leading causes of mortality worldwide. Cardiovascular diseases (CVDs) are responsible for nearly 33% of all global deaths and are considered the leading cause of disability-adjusted life years, accounting for 20%‒23% of the burden of disease.^1,2^ Studies have shown that 50% of CVD mortality and 80% of CVD global burden have occurred in low- and middle-income countries.^3^ In this regard, it seems that Iran may have the highest burden of CVDs in the Eastern Mediterranean Region.^4^ The main risk factors for CVDs include unhealthy lifestyles, hypertension (HTN), diabetes mellitus, hyperlipidemia, obesity, abdominal obesity, and smoking history.^4,5^

 HTN is particularly prevalent in the Iranian population and is associated with the highest risk of CVDs.^4,6^ In a national study conducted among 69 722 Iranian adults aged 25–65 years, more than half of the Iranian adult population was hypertensive or pre-hypertensive. Lack of awareness and a high uncontrolled rate of HTN are serious challenges in Iran.^4^ Lowering blood pressure is crucial to reducing the incidence of CVDs.^4,5^

 Excessive amounts of salt, the main source of sodium, come from the overconsumption of processed foods.^7,8^ Due to its low price and diverse properties, salt is one of the most widely used additives in the food industry.^8-10^ Based on available evidence, the Iranian population consumes much more salt than the recommended value of the World Health Organization (WHO).^7,11^ The mean daily salt intake was 9·52 g/d (95% confidence interval: 9.48, 9.56) in the Iranian population.^12^ Mohammadifard et al found that sodium and salt intake were about twice as high in Isfahan as the WHO recommended level.^13^ It is well known that excessive intake of salt is associated with adverse health outcomes such as HTN, heart attacks, and strokes.^3,4,14^ Reducing salt intake would be one of the more cost-effective measures to lower blood pressure and improve population health outcomes.^10,15,16^

**Figure 1:**
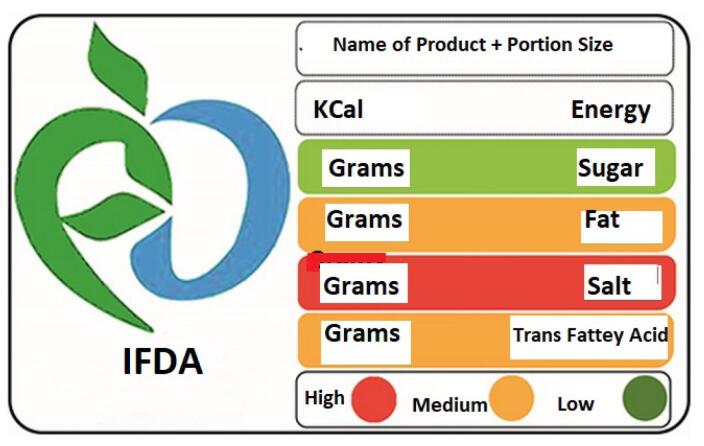
An Image of Traffic Light Labelling Inserted on the Food Package.

 Studies have demonstrated that food labelling plays a crucial role in promoting the reduction of salt intake by providing important information about the salt content of various food products for consumers. This condition enables consumers to make conscious decisions and select the intended food product with lower or free salt.^16-17^ Totally, the assessment of nutrition facts is sometimes accompanied by difficulties for consumers.^17^ For this purpose, the Iranian Food and Drug Organization implemented a national program in order to inform food consumers about the amount of salt. The strategy was the insertion of the traffic light on industrialized food labelling to represent the specification of NCD risk factors. It introduced the status of safe, risk, and cautious related to nutritional risk factors of NCDs with green, red, and green colors, respectively (Figure 1).^17^

 Nuts and seeds are rich sources of nutrients, including healthy fats, proteins, fiber, vitamins, and minerals. These groups of foodstuffs are included as significant components of the Iranian’s diet.^7,18,19^ Based on official reports and news, the production rate of some nuts and seeds, including pumpkin, peanut, sunflower, watermelon, almond, and pistachio, was 20 000, 30 000, 38 000, 70 000, 1 680 000, and 190 000 tons in 2022.^19^ Awareness among consumers about the content of salt in nuts and seeds is important to help them choose healthy products.^18^ The data and colour represented in the traffic light can be appropriate guidance for consumers at the time of purchase.^17^ The discrepancy in values was mentioned in labelling and measured in the laboratory, such as fat and trans fatty acids.^20,21^ The incorrect information on food labelling can mislead consumers into choosing suitable and healthy food.^21^ Considering that there is no data regarding examining the content of salt mentioned in traffic light labelling in Iran, the present study was conducted to determine the accuracy of the amount of salt in the traffic light of different packaged nuts and seeds available in the markets of Isfahan, Iran.

## Methods

 This cross-sectional study sought to measure the amount of salt in nuts (pistachios, peanuts, and almonds) and seeds (sunflower, pumpkins, and watermelons). A total of 53 samples from different producers were randomly purchased from several local markets in Isfahan, Iran. After collection, the samples were kept in the refrigerator until the analysis.

 Initially, the samples were milled, weighted (0.1 g), and transferred to vessels in order to add H_2_O_2_ and HNO_3_ for microwave digestion. Next, a 10% weight-volume solution of cesium chloride was prepared to be added to the digested samples. The sodium standards were prepared with concentrations of 2.5, 5, and 15 ppm. The blank solutions and sodium standards were injected into inductively coupled plasma-optical emission spectroscopy (ICP-OES; Varian Vista Pro, Australia) in order to obtain calibration curves. Afterward, all 53 digested samples were injected into the ICP-OES. The applied wavelength was 589.099 nm for the assessment of sodium amount regarding the baseline signals and interferences at selected lines found during the analysis. Finally, the achieved value of sodium was multiplied by 2.5 to calculate the quantity of salt. To prevent the effect of the organic matrix, the possibility of sample pollution, and losses of analytes, the containers were washed and subjected to nitric acid. Subsequently, deionized water was used for washing in order to prevent secondary contamination in the experiments.

 The Shapiro-Wilk test of normality was utilized to check for the assumption of normality of all quantitative variables. The amount of salt was reported as means ± standard deviations (SD). The independent sample T-test was employed to compare the mean difference of salt between the label and laboratory values. The obtained data were analyzed using Statistical Package for the Social Sciences (version 26, SPSS Inc., Chicago, IL, USA).

## Results

 The amount of salt was measured in 7, 8, 9, 9, 10, and 10 samples of pumpkin, pistachios, almond, sunflower, peanut, and watermelon, respectively. In three groups of samples, including sunflower seeds (5 out of 9 samples, 55.5%), pistachios (1 out of 8 samples, 12.5%), and pumpkin seeds (4 out of 7 samples, 57.1%), the salt laboratory value (10 out of 53 samples, 18%) was lower than the traffic light labelling value. In contrast, the laboratory analysis amount of salt in other samples was higher than the traffic light labelling amount. Overall, 82% of the studied products showed a discrepancy between the experimentally analyzed values of salt and the traffic light labelling.

 The data relating to the difference between the traffic light labelling and laboratory values are presented in Table 1. Varying levels of traffic light labelling value accuracy were observed in most of the samples. In the almond, pistachio, peanut, and watermelon groups, the average amount of laboratory value had a statistically significant difference with the label value (*P* < 0.05).

**Table 1:** The mean amount of salt (Mean ± SD) in traffic light labelling value and laboratory analysis (ppm) in different types of nuts and seeds

**Type of Sample**	**Traffic Light Labelling Value**		**Laboratory Value**		**Fold**	**Difference (95% CI)**	***P*** ** value**
	**Mean**	**SD**	**Mean**	**SD**			
Nuts							
Almond	911.1	581.9	5225.0	2804.4	5.73	4313.9 (2290.0, 6337.8)	0.001
Pistachio	1162.5	477.9	4382.2	2546.4	3.77	2843.3 (957.9, 4728.6)	0.006
Peanut	835.0	266.7	6579.1	1948.3	7.88	5744.1 (4437.6, 7050.5)	0.001
Seeds							
Watermelon	1430.0	590.8	6539.3	2824.5	4.57	5109.3 (3192.2, 7026.5)	0.001
Sunflower	2088.9	1331.8	4382.2	2546.4	2.10	357.3 (-1337.8, 2052.4)	0.661
Pumpkin	4171.4	4475.4	3094.1	2685.3	0.74	-1077.3 (-5375.4, 3220.7)	0.595

*Note*. SD: Standard deviation; CI: Confidence interval.

## Discussion

 In this study, the salt content reported in traffic light labelling of nuts and seeds was compared with measured values in the laboratory. Our findings revealed that the salt content in the most commonly packaged nuts and seeds (peanuts, 7.88 folds) was overall higher than that stated on the traffic light labelling. Excessive salt intake has been linked to numerous health problems, including high blood pressure, CVDs, and kidney problems.^12-14,22^ The discrepancy in salt content reported on traffic light labelling can undermine public health efforts to reduce salt consumption through clear and accurate labelling. Research has demonstrated that food labelling is considered a crucial public health tool aimed at providing consumers with information that influences their purchasing decisions. By having access to honest and accurate information, consumers can make healthier choices, which can have positive impacts on their overall health and well-being.^16,22-23^ An investigation in Iran demonstrated that 82.8% of consumers look at food label information when purchasing food products. Therefore, accurate traffic light labelling plays a crucial role in providing nutrition information to consumers.^17^ Moreover, accurate food labelling enables individuals to assess the nutritional value of a product, helping them make informed decisions about their diet and overall health. This information is particularly important for individuals with conditions such as diabetes, HTN, or heart disease who need to monitor and control their intake of specific nutrients such as saturated fats, sugar, or sodium.^24-26^

 Our study focused on the accuracy of the salt amount in the packaged nuts and seeds mentioned in food labelling, and discrepancies were observed between the information on traffic light labelling and laboratory-measured values. Salt was detected in all samples, exhibiting a range spanning from 2.1- to 7.9-fold higher concentrations compared to those reported on the respective traffic light labels. The range of concentrations was quite wide, with some samples containing up to 7-fold higher salt content than indicated on the traffic light labelling (Table 1). This discrepancy raises concerns about the accuracy of the information provided on food labels and suggests that consumers may unknowingly consume more salt than they read on the traffic light labelling. In addition, the observed inconsistencies between the labeled salt content and laboratory analysis results may mislead consumers and affect their dietary choices.

 In line with our findings, a total of 7,234 samples of packaged food items were examined in Canada, aiming to assess the sodium levels present in Canadian packaged foods in relation to the sodium guidelines established by the Canadian Ministry of Health. The findings revealed that 48.6% of the analyzed food items exceeded the permissible sodium level set by the guidelines, while 25% of all products exceeded the maximum allowable limit.^27^ A study was conducted in parts of Tehran, Iran, determining the salt content of 555 industrial and non-industrial products. It was reported that the mean salt content of both industrial (1.97/100 g) and non-industrial (2.16/100 g) nuts exceeded the Iranian standard (1.88/100 g).^7^

 The inaccurate labelling practices not only compromise consumer trust but also hinder the effectiveness of public health initiatives expected to reduce salt intake through clear and accurate labelling. Our finding emphasizes the need for stricter regulations and improving industry practices to ensure an accurate representation of salt content in traffic light labelling of food products. Urgent action is needed to safeguard public health interests, holding manufacturers accountable for providing truthful information. Additionally, due to the health risks associated with salt, determining the salt content of food products should be a regulation in the food industry.

**Highlights**

- The amount of sodium in the traffic light of labelling of nuts and seeds was examined in this study.
- The accuracy value was variable in most samples.
- The salt content of more than 80% of the studied samples had discrepancies with the values on the traffic light labelling.
- A significant difference in salt was observed between laboratory and label values in the almond, pistachio, peanut, and watermelon groups.

## Conclusion

 This study emphasizes a significant disparity between the salt content in traffic light labelling on packaged nut and seed products and the laboratory analysis results. Accurate labelling of salt content is essential for promoting healthy eating habits and enabling individuals to make informed choices about their diet. It seems that regulatory authorities should reexamine labelling guidelines and apply stricter compliance to certify accurate representation of salt content on traffic light labelling. The discrepancy between laboratory-measured salt content and reported values on food labels for packaged nuts highlights a critical issue within the food industry. Misleading consumers, compromising public health, and eroding trust in traffic light food labeling are severe consequences that necessitate immediate action. Collaboration between regulatory bodies, manufacturers, and consumer advocacy groups is vital to addressing this problem and providing accurate, transparent, and trustworthy nutritional information on food labels.

## Acknowledgments

 Our deepest thanks go to the staff of the laboratory of the School of Nutrition and Food Science at Isfahan University of Medical Sciences for their precious support at different stages of this study.

## Authors’ Contribution

**Conceptualization:** Fatemeh Shirani, Zahra Esfandiari.

**Data curation:** Seyedeh Mahsa Khodaei, Raziyeh Eshaghi, Zahra Esfandiari.

**Formal analysis:** Fatemeh Shirani, Mojtaba Akbari.

**Funding acquisition:** Mansour Siavash.

**Methodology:** Fatemeh Shirani, Mojtaba Akbari, Zahra Esfandiari.

**Project administration:** Mansour Siavash.

**Resources:** Fatemeh Shirani, Zahra Esfandiari.

**Software:** Fatemeh Shirani, Mojtaba Akbari.

**Supervision:** Mansour Siavash.

**Validation:** Fatemeh Shirani, Zahra Esfandiari.

**Visualization:** Fatemeh Shirani, Zahra Esfandiari.

**Writing–original draft: **Fatemeh Shirani, Mojtaba Akbari, Zahra Esfandiari.

**Writing–review & editing:** Fatemeh Shirani, Zahra Esfandiari.

## Competing Interests

 The authors are required to disclose financial or non-financial interests that are directly or indirectly related to the work submitted for publication.

## Ethical Approval

 The study was approved by the Research Ethics Committees of the “Alzahra Research Centers”, Isfahan University of Medical Sciences, with specification number IR.ARI.MUI.REC.1400.090.

## Funding

 This work was supported by the Vice-chancellor of Research and Technology of Isfahan University of Medical Sciences (Grant number 2400237).
